# A battery-less implantable glucose sensor based on electrical impedance spectroscopy

**DOI:** 10.1038/s41598-023-45154-8

**Published:** 2023-10-23

**Authors:** Stig Ollmar, Alejandro Fernandez Schrunder, Ulrik Birgersson, Tomas Kristoffersson, Ana Rusu, Elina Thorsson, Patricia Hedenqvist, Elin Manell, Anneli Rydén, Marianne Jensen-Waern, Saul Rodriguez

**Affiliations:** 1 Department of Clinical Science, Intervention and Technology, Karolinska Institute, Stockholm, Sweden; 2 School of Electrical Engineering and Computer Science, KTH Royal Institute of Technology, 16440 Kista, Sweden; 3 Prevas AB, Stockholm, Sweden; 4 Pathology Unit, Department of Biomedical Science and Veterinary Public Health, Swedish University of Agricultural Sciences, Uppsala, Sweden; 5 Department of Clinical Sciences, Swedish University of Agricultural Sciences, Uppsala, Sweden

**Keywords:** Preclinical research, Translational research

## Abstract

The ability to perform accurate continuous glucose monitoring without blood sampling has revolutionised the management of diabetes. Newer methods that can allow measurements during longer periods are necessary to substantially improve patients’ quality of life. This paper presents an alternative method for glucose monitoring which is based on electrical impedance spectroscopy. A battery-less implantable bioimpedance spectroscope was designed, built, and used in an in vivo study on pigs. After a recovery period of 14 days post surgery, a total of 236 subcutaneous bioimpedance measurements obtained from intravenous glucose tolerance tests, with glucose concentration ranges between 77.4 and 523.8 mg/dL, were analyzed. The results show that glucose concentrations estimated by subcutaneous bioimpedance measurements correlate very well to the blood glucose reference values. The pigs were clinically healthy throughout the study, and the postmortem examinations revealed no signs of adverse effects related to the sensor. The implantation of the sensor requires minor surgery. The implant, being externally powered, could in principle last indefinitely. These encouraging results demonstrate the potential of the bioimpedance method to be used in future continuous glucose monitoring systems.

## Introduction

Diabetes mellitus (DM) is a chronic disease characterized by hyperglycemia and altered metabolism. The number of patients suffering from DM is increasing globally, and in 2020, WHO calculated that 422 million people suffered from DM. The disease is the seventh most common cause of death^1^. Better treatment methods and blood glucose (BG) control are necessary to improve quality of life and life expectancy of the patients. Blood glucose can be measured by analyzing a drop of blood from the fingertip with a test strip, e.g. Accu-Chek (Roche Diagnostics, Basel, Switzerland) or by using a device for continuous glucose monitoring (CGM). CGM provides both an instant BG value in combination with a BG trend which allows for proactive diabetes management rather than reactive.

In recent years, a variety of CGM have emerged on the market^2^ with the most frequently used being based on the enzymatic electrochemical reaction with glucose^3,4^ and the second most based on a fluorescence sensor that uses a polymer which alters the fluorescence intensity when bound with glucose^5^.

Even though todays CGMs offer patients with DM an ability to manage glucose levels in an unprecedented way there still remain some shortcomings. First, todays CGMs have a limited lifespan either due to the depletion of the glucose oxidase enzyme after 10–14 days or through slow degeneration of the fluorescence-based sensor by reactive oxygen species after up to 6 months of use. Second, CGMs may cause infection and pain over time either due to the small incision and opening to the dermis or due to the incision made during placement of the fluorescence-based sensor under the dermis. Additionally, the adhesive used to attach the monitor may cause pain, and skin irritation over time. Third, there is always an inherent risk of dislodging the electrochemical sensor which may result in having to use finger pricking as a replacement until a new sensor can be applied.

A different way of estimating blood glucose levels appeared in a serendipitous way in the early 90’s where covariation between BG and dielectric properties were observed during skin moisturization measurement^6^. Following this observation, a considerable number of studies have been conducted to try using skin bio-impedance measurements in glucose monitoring applications. However, it was found that bioimpedance is affected by several factors^7–19^ of which many have diurnal variations such as skin pH^20^ and it was concluded that designing a reliable sensor based on electrical measurements of intact skin may be very difficult, if at all possible, which led us to investigate the potential of implantable sensors. All required electronic components of such an implantable sensor were developed by the authors and tested *in vitro* on freshly excised organs of sheep^21^ and also on rats in vivo. For in vivo testing, rats turned out to be a poor model, not only due to their size but also their remarkable body movement ability. Therefore, in this study pigs were chosen due to their close anatomical and physiological resemblance to humans^22^ and that they are well-suited for intravenous glucose tolerance tests^23^.

The objectives of this study are to establish proof of principle for an implantable glucose sensor based on bio-impedance spectroscopy, and to validate biocompatibility and safety of such a device. This working proof of concept prototype can be further improved, e.g. smaller dimensions in order to be more suitable for clinical use.

## Results

### Experimental design of the porcine studies

After the acclimatization and training period, all animals were fitted with indwelling catheters (SIL-C70 with rounded tip; Instec Solomon, Plymouth, Meeting PA, USA) into *vena jugularis* under aseptic conditions and general anesthesia. Anesthesia was induced with tiletamin-zolazepam (Zoletil forte^®^ vet; Virbac, Carros, France) mixed with medetomidine (Domitor^®^ vet; Orion, Espoo, Finland) intramuscularly (IM). After endotracheal intubation anesthesia was maintained with isoflurane (IsoFlo^®^ vet.; Orion Pharma Animal Health, Sweden) in an oxygen/air mixture. For analgesia, buprenorphine was given IM pre- and postoperatively. For dosages see^24^. Circulatory and respiratory parameters were continuously monitored under anesthesia. The catheters were surgically implanted, tunnelled subcutaneously and exteriorised at the back between scapulas^25^. Also, catheters were inserted into *vena auricularis* by minimally invasive Seldinger technique.

**Figure 1:**
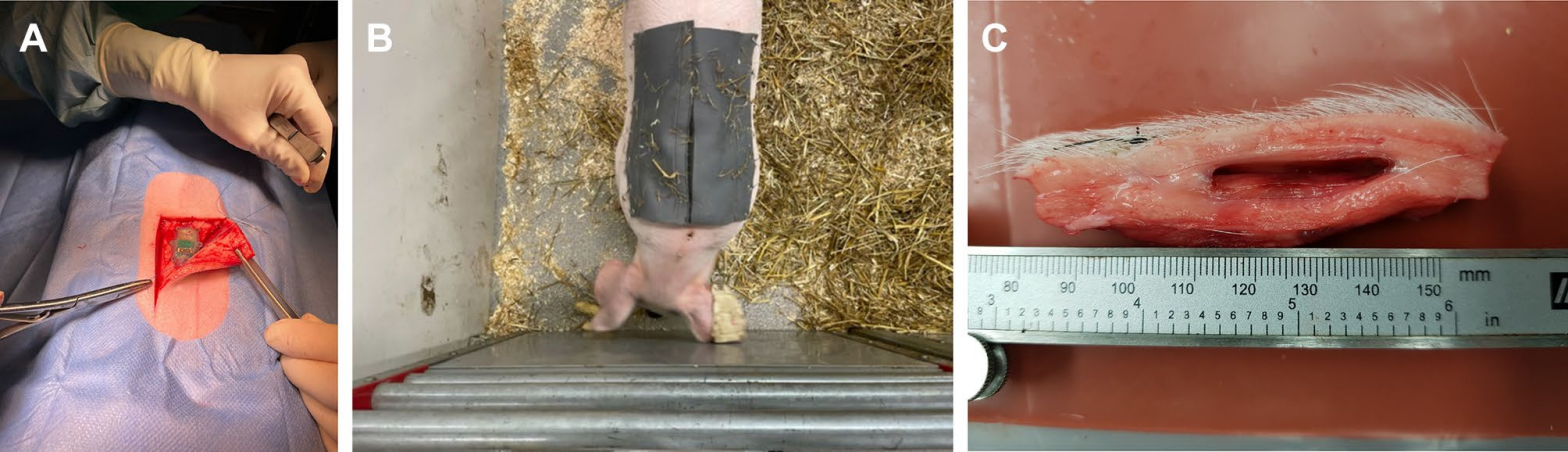
(**A**) Sensor implantation. (**B**) Pig with canvas holding a reader. (**C**) Position of device in subcutis, device removed.

The sensors were autoclaved in EtO before inserted into the pigs. An L-shaped incision was made in the epidermis and the biosensor was placed 5 cm caudal to the catheter as shown in Fig. 1A. The sensors were sutured onto the fascia and the skin was closed with single VICRYL^®^ sutures. Finally, to protect the sensor and catheter, a canvas cover with a pocket for the reader was sutured onto the pig’s back as shown in Fig. 1B. Heart rate, respiratory rate, oxygen saturation and body temperature were monitored until the pigs regained consciousness. Additional oxygen was given if needed. All animals recovered quickly after the surgery and started to eat within 6 hours. Blood samples were collected in serum and EDTA tubes. Analyses of total and differential white blood cell counts and hematology (EPK, Hb, EVF, MCV, MCHC, reticulocytes, thrombocytes) were performed. Serum samples were analyzed for aspartate amino transferase (ASAT), alanine amino transferase (ALAT), -glutamyltransferase (GT), glutamate dehydrogenase (GLDH) and creatinine. The analyses are validated for pigs and were conducted at the department of Clinical Pathology, SLU, Uppsala. The results from hematology and clinical chemistry were all within the reference values. The pigs received antimicrobials for three days. Clinical appearance was assessed daily throughout the studies and a thorough clinical examination was performed twice a week. The animals were weighed twice a week and daily weight gain calculated. Their weight gain was similar to that of SPF pigs kept at Lövsta university herd, Uppsala.

Four pilot plus 10 pigs fitted with the first prototype sensor were euthanized with intravenous pentobarbital sodium (Allfatal vet. Omnidea, Sweden) 17–20 days after implantation of the sensor, and two animals after 44 days. Nine pigs with the second prototype sensor were euthanized 27–28 days after the implantation.

A full post *postmortem* examination including a detailed examination of the site of implantation, was performed by a veterinary pathologist (Fig. 1C). Autopsy was routinely performed on body warm carcasses. Representative samples of eligible tissues and organs were collected and fixed in 10% neutral buffered formalin for > 24 h, paraffin wax embedded, sectioned at 4 μm and stained with hematoxylin and eosin (HE). Selected tissue specimen were stained with tissue marking dye (Thermo-Shandon marking dye) prior to embedding. All sections were evaluated using light microscopy. Capsule formation surrounding subcutaneous implants was assessed using calipers and/or measure tool in NIS-elements software imaging by Nikon.

### Intravenous glucose tolerance test (IVGTT)

**Figure 2:**
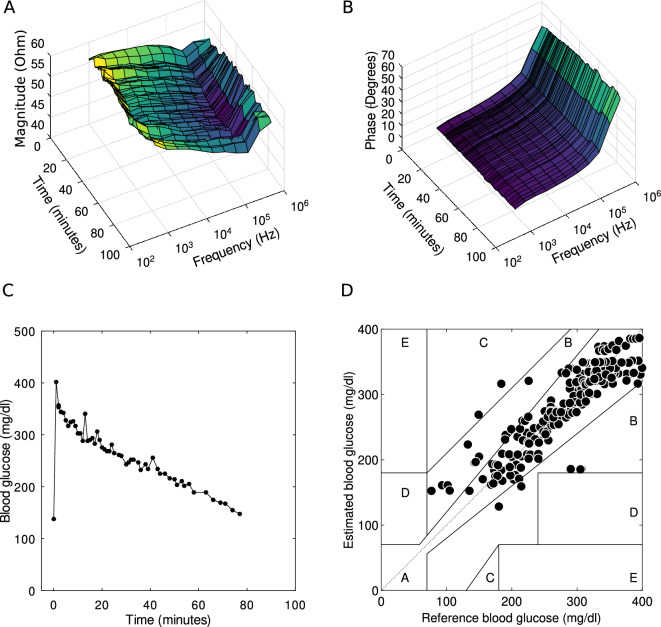
Example of (**A**) measured magnitude spectra and (**B**) measured phase spectra over time. (**C**) Example of blood glucose curve during IVGTT in pigs. (**D**) Estimated blood glucose based on electrical bio-impedance measurements versus reference blood glucose measurements, presented in a Clarke Error Grid to show clinical significance.

Two weeks after the surgery, the pigs underwent IVGTT. After at least five hours of fasting, glucose (Glucose Fresenius Kabi, 500 mg/ml, Fresenius Kabi, Halden Norway) was infused intravenously at a dose of 0.5 g/kg bodyweight during 60 s. Blood samples were collected before, and frequently up to 4 h after glucose infusion. Blood glucose concentration was measured immediately after sampling with test strips (Accu-Chek, Roche Diagnostics, Basel, Switzerland; validated for porcine blood at the Department of Clinical Chemistry, SLU, Uppsala, Sweden). At the same time that glucose concentration was being measured, bioimpedance measurements were registered by using the external reader. Each measurement captured 11 complex impedance data points spanning from 1 kHz to 1 MHz and that were acquired as frequent as once per minute. Representative outcomes, magnitude and phase spectra, along with measured blood glucose concentrations, are illustrated in Fig. 2A–C respectively. The impedance spectra for the entire cohort of porcine subjects can be referred to in Supplementary Fig. S2. It is noteworthy that the impedance spectra across all subjects exhibited consistent patterns, characterized by diminished resistive values at lower frequencies transitioning to capacitive impedance behaviours at elevated frequencies. In Supplementary Fig. S1, the measured blood glucose concentrations are presented with the EIS-derived estimates of glucose. The blood glucose concentrations ranged from 77.4 to 523.8 mg/dL.

A total of 286 blood samples and 236 impedance measurements were taken from four pigs during six IVGTT runs.

### Glucose correlation

The reference glucose concentrations and the estimated glucose concentrations measured with the bioimpedance spectrometer are shown in Fig. 2D. The plot includes a Clarke grid which provides information about consequences of any errors in the delivered measurement value. Zones A and B represent very low risk and are good enough for a medical device requiring no intervention by neither the patient nor medical personnel, while zone E suggests an intervention opposite to what is needed which can be potentially lethal. The data used to create Fig. 2D is available as Supplementary Fig. S1. Only 2 points out of 236 fell outside zones A or B, and those two fell in zone C. It is believed that the deviations from ideal (zone A) are mainly caused by body motions of the animal and the rapid glucose changes immediately after start of IVGTT not in exact pace with the blood sampling, see section “Discussion”.

For all IVGTTs, the frequencies that turned out to be the most beneficial ones are found in between 10 and 100 kHz, which happen to include the most informative range from the Beta dispersion. The other frequencies allow for noise identification and in extension for a potential improvement in accuracy. As anticipated, adjacent frequencies exhibited comparable results. This is attributed to the cross-correlation of the measured impedance, e.g., 10 kHz and 20 kHz convey to a large extent overlapping information.

### Animal response

In general, the animals recovered quickly after surgery and were healthy throughout the study. Some animals showed a decreased general appearance because of inflammation or infection induced by the jugular catheter. These animals were promptly treated with antibiotics and clinically recovered after medication. This diagnosis was confirmed at postmortem examination.

Gross pathology examination revealed all pigs to be within average body condition and without systemic disease. Based on macroscopic and microscopic observations, there were no general acute or delayed toxicity after subcutaneous implantation of PLA biosensor or silicon biosensor.

A capsule formation consisting of connective tissue was observed around the device. In most animals this capsule was <1–1.5 mm thick. In a few animals, the capsule was up to 4 mm thick in the ventral position.

Autopsies of 10 pigs fitted with the PLA prototype were performed at the Division of Pathology, Department of Biomedical Sciences and Veterinary Public Health, Swedish University of Agricultural Sciences, at 17d (2 individuals), 20d (6 individuals) and 44d (2 individuals) post-surgery. Autopsies of 9 pigs fitted with the silicon prototype were performed as described for the PLA cohort.

## Methods

### 4-terminal impedance measurement technique

Bioimpedance can be measured using 2-terminal measurement systems^26^. While this method requires only two electrodes, it has a significant limitation, especially for implantable settings. Since implants are typically small, the electrodes need to be correspondingly small, leading to high impedance. At the same time, subdermal tissues exhibit high electrical conductivity, resulting in low bioimpedance values. Such factors complicate the extraction of electrode impedance from the measurement, a complication that is further intensified by electrode mismatches. This issue stems from the 2-terminal method’s reliance on the same electrodes for both electrical excitation injection and response sensing.

A way to address this issue is employing a 4-terminal method that utilizes two specific electrodes for electrical excitation, typically an AC current, and two distinct electrodes for sensing the resultant AC voltage across the tissue^27^. Given that voltage-sensing circuits exhibit high input impedance, current doesn’t traverse the sensing electrode impedances, thus excluding them from measurements and enabling bioimpedance measurements with sub-1 Ohm errors. The primary constraint of the 4-terminal approach is the requirement of two extra electrodes. Given the ongoing miniaturization of electronic circuits, the implant’s minimum size becomes governed by electrode dimensions and configuration rather than electronic constraints. Nonetheless, in terms of measurement precision, the 4-terminal technique was deemed optimal for the implantable bioimpedance sensors.

### Implantable bioimpedance sensor

Latest advances in microelectronics have made possible the integration and miniaturization of bioimpedance sensors for a wide variety of applications. Some of the state-of-the-art integrated solutions for bioimpedance measurements are found in^28–33^. In addition, a commercial product for bio-impedance spectroscopy has been recently released^34^.

**Figure 3:**
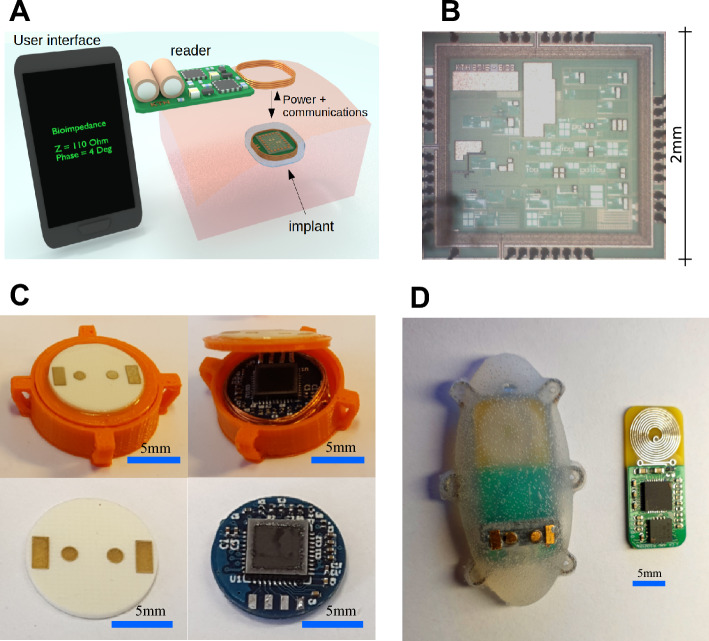
Implantable bio-impedance spectroscopy system.

The bioimpedance sensor used in this work is based on an improved version of a batteryless implantable bio-impedance sensor previously reported by the authors^21,35^. The complete system is depicted in Fig. 3A and consists of an implantable device, an external reader, and a user interface running either on a computer or a mobile device. The implantable device is a 4-terminal impedance measurement system that is externally powered by the external reader by using inductive coupling at a frequency of 10 MHz. The external reader is able to power-up the implant up to a distance of 4 cm. Communication from the reader to the implant is achieved by employing ASK modulation of the 10 MHz signal. Communication from the implant to the reader is achieved by using backscattering modulation. The reader communicates with a user software running in a computer or mobile device by using Bluetooth.

A new application specific integrated circuit (ASIC), shown in Fig. 3B, was designed in a 0.18 μm CMOS process. This ASIC includes a fully integrated communications module, a 32-step sinusoidal generator instead of 8-step (much lower harmonic content), a 64X PLL instead of 32X PLL, and 9 possible gains options which allows measurements from 100 $\Omega$ to 1 M$\Omega$ with 1% precision. The injected current is programmable with a range from 123 nA to 10 μA. Its dimensions are approximately 2 mm $\times$ 2 mm, and it consumes 274 μA from an internal 1.8 V power supply. The ASIC is able to measure complex impedances from 1 kHz to 1 MHz in 11 logarithmic steps. Several samples of this ASIC were packaged in QFN-40 packages and mounted in two different implantable prototypes before being used in the animal studies.

The first prototype is shown in Fig. 3C, and it has a cylindrical shape of 15 mm in diameter and 5 mm height. Four fixation points were added to the border of the sensor in order to allow for suturing it to the soft tissue. Fig. 3C also shows the sensor’s electronics including a circular air core inductor inside the module. This module was 3D printed in polylactide (PLA) material. The gold electrodes were built on a ROGERS substrate using ENIG process. The rectangular electrodes, designated for current injection, measure 1.4 mm $\times$ 3 mm. The internal circular electrodes, purposed for voltage detection, have a diameter of 1.4 mm. The distances from the chip’s center to the centers of these electrodes are 1.9 mm and 4.3 mm, respectively. The packaged ASIC was mounted on the top of a 4-layer FR4 PCB. At the bottom of this PCB there is an 8-bit microcontroller. Both PCBs have a diameter of 11 mm.

The PCB and implantable module for the second prototype are shown in Fig. 3D. The air core inductor was replaced by a planar circular inductor built on the same RF4 PCB that holds the ASIC and the 8-bit microcontroller. This was done in order to reduce losses that appeared in the first prototype due to the electronics being located in the center of the air core inductor. The PCB was mounted in a holding frame which also holds the 4 gold plated electrodes. The dimensions of the electrodes remain consistent with those of the initial prototype. This structure was coated with Parylene before being molded in medical grade silicon, MED2-4420 (NuSil). The implantable module measures 40 mm $\times$ 18 mm $\times$ 6 mm. Six fixation points were added at the border for suturing purposes.

### Animals and housing conditions

The animal studies were approved by Uppsala Animal Ethics Committee (Ethical permission Dnr 5.8.18-19258/2017) and SLU, UA (Dnr 2021.4.1-2834). The guidelines in Directive 2010/63/EU “on the protection of animals used for scientific purposes” were followed and the use of animals reported in accordance with ARRIVE guidelines.

Conventional domestic pigs (n = 25; Yorkshire $\times$ Hampshire; females and castrates) with certified specific pathogen free status were obtained from the University herd, Lövsta, SLU, Uppsala, Sweden. At arrival at the Dept. of Clinical Sciences, VHC, SLU, Uppsala, the pigs were 8 weeks old. The pigs were kept in individual pens measuring approximately 3 m$^2$ within sight and sound of each other. A 12:12h light/dark schedule was used and an infrared lamp (24h) was placed in the corner of each pen. As bedding, straw and wood shavings were used. The pens were cleaned twice daily. The room temperature was 18 ± 2 $^\circ$C. Twice daily, the pigs were fed commercial pig feed (Solo 3330, Lantmännen, Sweden) the amount according to SLU regimen for growing pigs. Water was provided ad libitum. All animals had an acclimatization and socializing period of at least 14 days before taken into the study. During this period, the pigs were trained to accept clinical examinations and accept touching and palpation of the ears and back, as preparation for stress-free blood sampling from indwelling catheters placed in the auricular and jugular veins. Further, the animals were accustomed to a reader dummy kept in a canvas pocket, later sutured onto the skin. They were also habituated to the sound of Velcro used for opening and closing of the canvas pocket. Each pig was trained daily for 15 min.

### Signal processing and statistics

For each IVGTT, the magnitudes and phase angles of the electrical impedance spectra underwent a median filtering process over time, using a 5 min rolling median. This filtering is applied to reduce the influence of artifacts in the impedance measurements. The timestamps of the impedance measurements were aligned to the timestamps of the blood glucose measurements by identifying the closest time point. The relationship between the measured blood glucose and both the magnitude and phase at every frequency is evaluated. The optimal frequency is determined based on the Pearson correlation. Thereafter, a standard linear regression was used, linking the impedance parameter with the strongest correlation to the measured blood glucose, to estimate the glucose levels. The measured blood glucose versus impedance estimated glucose concentrations were plotted in Clarke error grids, and the number of observations in the different zones were calculated. The data of each subject and occasion were evaluated separately. Data analysis was made in python programming language; filtering, data alignment and correlation analyses were made using Pandas, the linear regression using the scikit-learn package, and the Clarke error grid analysis was made using in-house code. For details see the section on Glucose Correlation.

## Discussion

This study shows that the blood glucose concentration estimated by a subcutaneously placed bioimpedance-sensor is strongly correlated with reference blood glucose values obtained from a commercial glucose meter. The data are clinically relevant and show that the bioimpedance spectroscopy method has the potential of being used in future CGM systems. Two important advantages of such a bioimpedance based implantable sensor are that it has no components that significantly degrade over time and it does not require any battery replacement. In principle, this would mean that only one implantable sensor would be needed to conduct a lifetime of glucose measurements for a patient. However, five questions still remain to be answered.

First, what potential physiological mechanisms might link tissue impedance with blood glucose concentrations? The exact underlying mechanisms in which variations of blood glucose concentrations produce variations of impedance are not yet understood. One plausible explanation is related to the chain of chemical reactions involving glucose (glycolysis) as part of the production of adenosine triphosphate (ATP). The release of ions H$^+$ and NAD$^+$, and more specifically the change of their ionic concentrations could affect the electrical conductivity of the tissues in a measurable level. The concept of indirectly measuring electrical conductivity in order to estimate ionic variations due to anaerobic respiration in glycolysis has been for instance proposed in order to estimate fetal hypoxia^36^. Future studies should aim to investigate the specific physiological processes that link changes in tissue properties to blood glucose levels. An understanding of these mechanisms will enhance the accuracy and reliability of glucose estimation using electrical impedance spectroscopy.

Second, is the device safe to use over time? The implantable device is based on proven biocompatible materials such as PLA, medical graded silicon, gold electrodes, as well as parylene in the device coating. The combination of these materials in our study did not induce any adverse effects such as acute or delayed toxicity in pigs which indicates that it is safe to use during the time period studied (up to 44 days). However, possible adverse reactions should be studied over a longer period of time.

Third, how is the glucose sensor impacted by everyday life and will the bioimpedance measurement change over time? Movement of the electrode system is known to interfere with electrical impedance measurements. To reduce interference from body motions, the size of the device was optimized, in particular the size of the electrode system and its geometry, in relation to the anatomical location of the implanted sensor. Therefore, an elliptic shape was chosen for the sensor with the electrode system placed flush with the convex surface. Additionally, simulations based on prior studies^37–39^, were conducted with a transition from 2-point to 4-point impedance measurements considering various positions of a small amount of leaked and accumulated body liquid in the vicinity of the electrodes. The simulations utilized the geometry and size of the sensor as previously outlined and typical literature values of conductivity of body liquids and tissue^21,40^. The simulation showed that in the worst case, the measured impedance may easily deviate one order of magnitude from the ideal case. Thus, good adherence between the surrounding tissue and the sensor surface is of paramount importance which was further accounted for by the addition of fixation eyelets on the side of the sensor. A more detailed overview is presented in Supplementary material S3.

The penetration of the test current into the tissue is determined by the distance between the electrical current injection electrodes. The effective penetration depth will be about half the distance between those electrodes. A shorter distance would reduce effects related to body motions, but if made too short, the measurement will not extend outside the fibrous encapsulation which is formed around the sensor. The present sensor size and design is a good compromise between such considerations. Our pigs were not sedated in order not to alter normal metabolism. We cuddled the pigs with a brush and offered them small amounts of food in order to keep them calm, but sometimes they rolled around in the pen, exposing the sensor location to excess movement which would give rise to much larger spread of data points than in the intended human use.

In this sense, a very important question to answer is whether the bioimpedance method requires frequent calibration against e.g. Accu-Chek or other calibrated and validated instruments such as in^5^ or if the encapsulation process disturbs repeatable bioimpedance measurements over longer periods of time. Future studies should aim to investigate the device’s performance over time against a commercially available CGM alongside a single reference blood glucose measurement to ascertain the frequency of calibration necessary.

Finally, is the size of the implant applicable for human use? The proof-of-concept prototype as depicted in Fig. 3C,D shows a rather large device that may not be amenable for human implantation. However, further miniaturization of the sensor is possible by minimizing the footprint of its components. This can be done in several ways, for instance, by using advanced packaging such as Wafer Level Chip Scale Package (WLCSP), or directly bonding the ASIC to the PCB by using chip-on-board (COB). In addition, further miniaturization is possible by integrating various passive components inside the ASIC that are currently external. This is possible by redesigning the ASIC using more modern CMOS technologies which could allow for a device less than half the size as the current prototype while maintaining the electrode size.

## Conclusion

For the first time, we have demonstrated that an implanted electrical-impedance-spectroscopy sensing device may have the potential to accurately estimate blood glucose. No adverse events or adverse device effect were encountered during the study, which opens the potential for bioimpedance-sensor in future CGM systems. The next step will be to compare performance of our prototype sensor with a CGM in humans.

### Supplementary Information

Supplementary Information.

## Data Availability

All the data are contained in this paper. Saul Rodriguez, the corresponding author of this manuscript, will supply more information upon reasonable request.
