# Supplementary material for: A battery-less implantable glucose sensor based on electrical impedance spectroscopy
Source: Sci Rep. 2023 Oct 23;13:18122. doi: 10.1038/s41598-023-45154-8 (PMC10593792; doi:10.1038/s41598-023-45154-8)
Supplement: Supplementary file 1 — Supplementary Information. [file 41598_2023_45154_MOESM1_ESM.pdf]

SUPPLEMENTARY INFORMATION

S1 Reference and estimate blood glucose concentrations

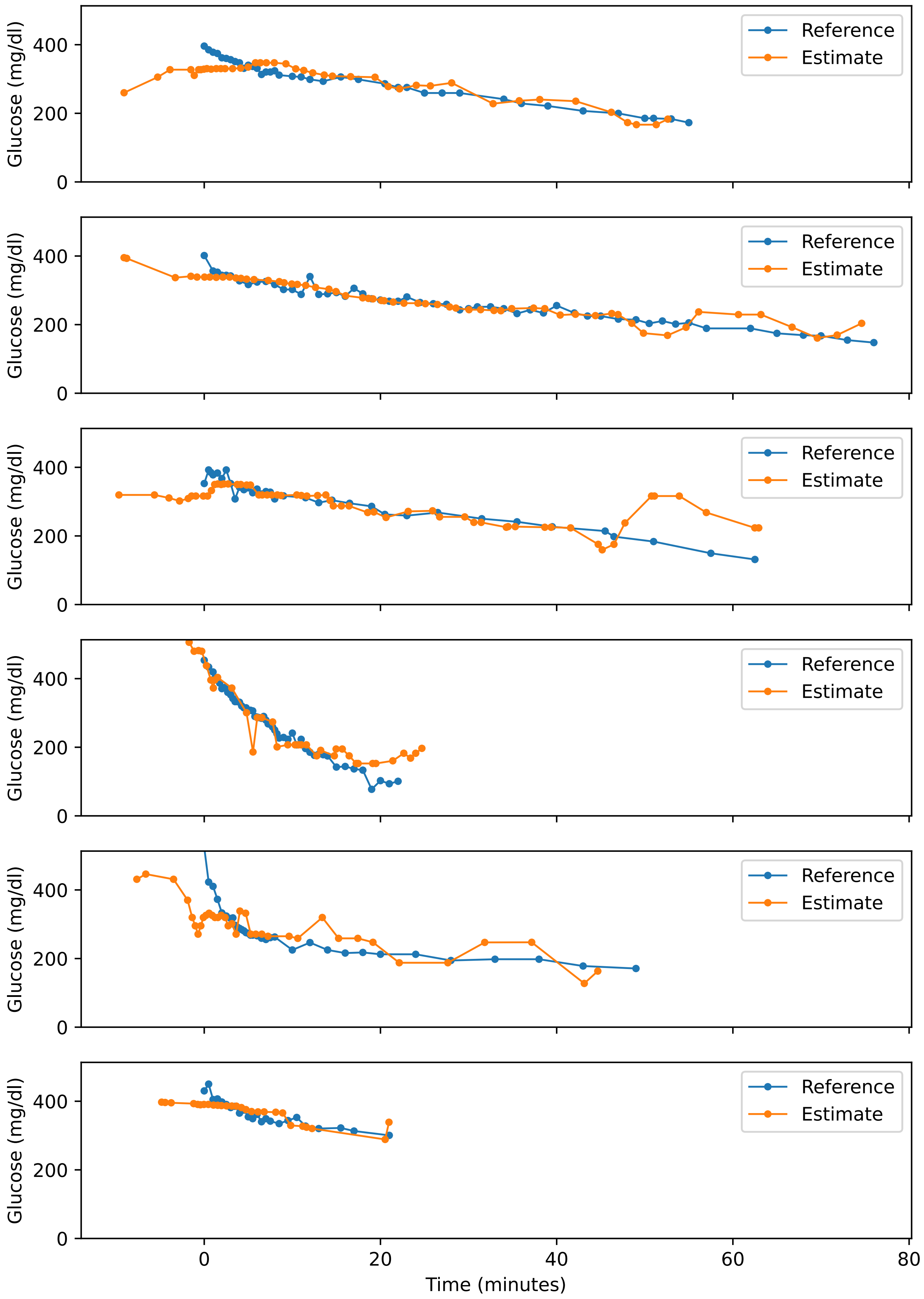

Figure S1. Reference blood glucose concentrations obtained using a commercial glucose meter and estimate blood glucose concentrations obtained by using bioimpedance measurements

S2 Measured in vivo impedance spectra

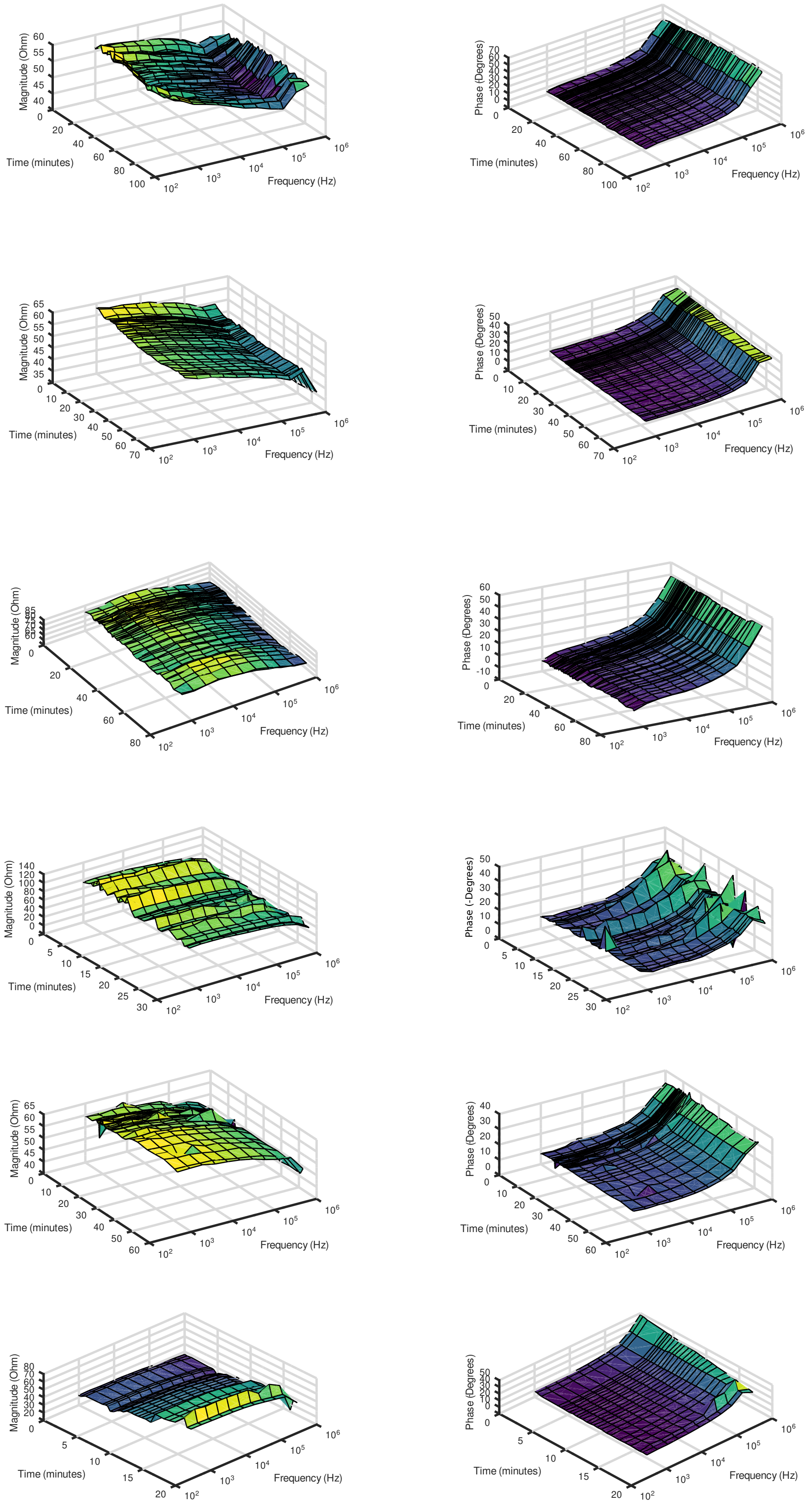

Figure S2. Measured Impedance and Phase spectra obtained using the implantable sensor

S3 Electrode Size and Simulation Cases

The transition from the 2-point to the 4-point impedance measurement method was based on prior studies. Using the sensor's dimensions and the electrode-tissue relationship shown in figure S3A-B, along with recognized literature values for body fluid and tissue conductivity (with liver tissue presented here), simulations were conducted to assess potential discrepancies arising from small leakages or build-up of bodily fluids around the electrodes.

Results, displayed in Figure S3C, revealed that the worst-case impedance could deviate tenfold from the ideal scenario. Hence, ensuring a tight bond between the tissue and the sensor surface is crucial, leading to the inclusion of fixation eyelets on the sensor's side.

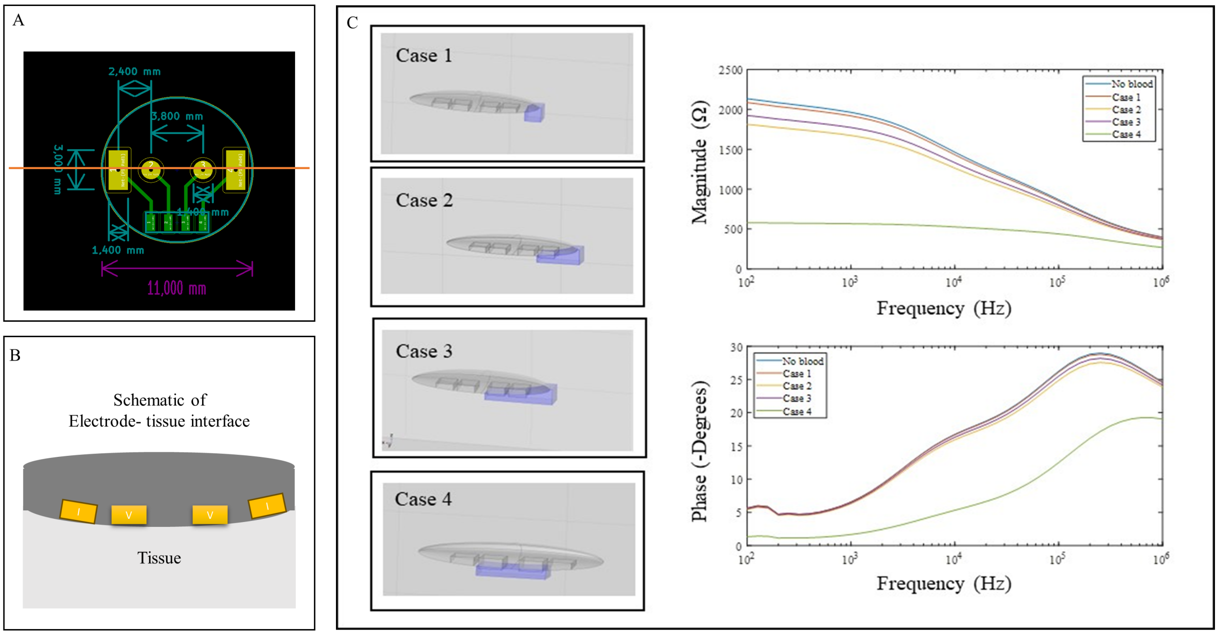

Figure S3. (A) Details of electrode sizes. (B) Schematic overview of electrode-tissue interface. (C) Electrical impedance results for four scenarios (Case 1-4) showing the impact of different blood positions beneath the electrode, compared to a blood-free situation, along with the simulated magnitude and phase values spanning frequencies from 1kHz to 1MHz.
